# Coexistence of low-grade pulmonary mucinous epithelioid carcinoma and metastatic adrenal sarcomatoid carcinoma: a rare case report with BRAF p.V600E-driven molecular insights and clinical challenges

**DOI:** 10.3389/fonc.2025.1564472

**Published:** 2025-08-15

**Authors:** Guangyan Xu, Fan Zou, Yuanbo Lan

**Affiliations:** Department of Respiratory and Critical Care Medicine, Affiliated Hospital of Zunyi Medical University, Zunyi, China

**Keywords:** pulmonary mucinous epithelioid carcinoma (PMEC), adrenocortical sarcomatoid carcinoma (ASC), metastasis, imaging features, immunohistochemistry, next-generation sequencing (NGS)

## Abstract

**Introduction:**

Pulmonary mucinous epithelioid carcinoma (PMEC) is a rare malignancy that typically progresses slowly and has a favorable prognosis. In contrast, adrenal sarcomatoid carcinoma (ASC) is an aggressive and uncommon cancer with poor outcomes. The coexistence of low-grade PMEC and metastatic ASC is exceedingly rare and presents unique clinical challenges, with limited treatment options and poor prognosis. This case report highlights the diagnosis and management of a patient with long-term, slow-progressing low-grade PMEC and rapidly progressing metastatic ASC.

**Case presentation:**

A 44-year-old male with a 20-year history of intermittent respiratory symptoms developed abdominal pain and imaging findings indicative of adrenal metastasis and multiple bone metastases. Initial diagnosis through CT and PET-CT scans raised suspicion for pulmonary tumors, and subsequent biopsies confirmed low-grade PMEC in the lungs. In 2023, further diagnostic work revealed a sarcomatoid carcinoma (SC) in the left adrenal gland. Molecular testing revealed BRAF p.V600E mutations across lung, adrenal, and plasma samples, providing critical insight into the nature of the metastatic spread. Despite treatment with molecular therapy (dabrafenib + trametinib) and radiotherapy, the patient’s conditioan deteriorated rapidly, and he passed away in September 2023.

**Discussion:**

This rare case underscores the importance of the BRAF p.V600E mutation in guiding therapy in cases of coexisting PMEC and ASC. The consistent presence of BRAF mutations in lung, adrenal, and plasma samples provided molecular evidence of the metastatic process, offering guidance for targeted therapy. Despite the potential of molecular therapy, the limited treatment efficacy suggests that further research is needed to better identify patient populations that may benefit from targeted therapies for advanced PMEC. BRAF mutations play a significant role in treatment decision-making and should be considered in clinical practice for these complex cases.

**Conclusion:**

This case highlights the complexity of diagnosing and treating coexisting low-grade PMEC and metastatic ASC, with the BRAF p.V600E mutation offering valuable molecular insights for therapy. Treatment strategies should be personalized, and future studies are needed to refine therapeutic approaches for such complex cases.

## Introduction

Pulmonary mucinous epithelioid carcinoma (PMEC) is a rare malignancy that arises from Kulchitsky cells in the submucosal glands of the trachea and bronchi. Low-grade PMEC generally progresses slowly and is associated with a favorable prognosis. Adrenal sarcomatoid carcinoma (ASC), on the other hand, is an uncommon and aggressive malignancy, with a complex pathogenesis. Its symptoms are often nonspecific, leading to delayed diagnosis, and it is associated with a poor prognosis. The coexistence of low-grade PMEC and metastatic ASC is extremely rare, and the condition typically deteriorates rapidly. Treatment options are limited, and prognosis remains very poor. This case report describes a patient with long-term, slow-progressing low-grade PMEC and rapidly progressing metastatic ASC, diagnosed through pathology at the Affiliated Hospital of Zunyi Medical University. Additionally, a review of the literature is provided to enhance clinicians’ understanding of this rare and challenging clinical entity.

## Clinical data

**Figure 1:**
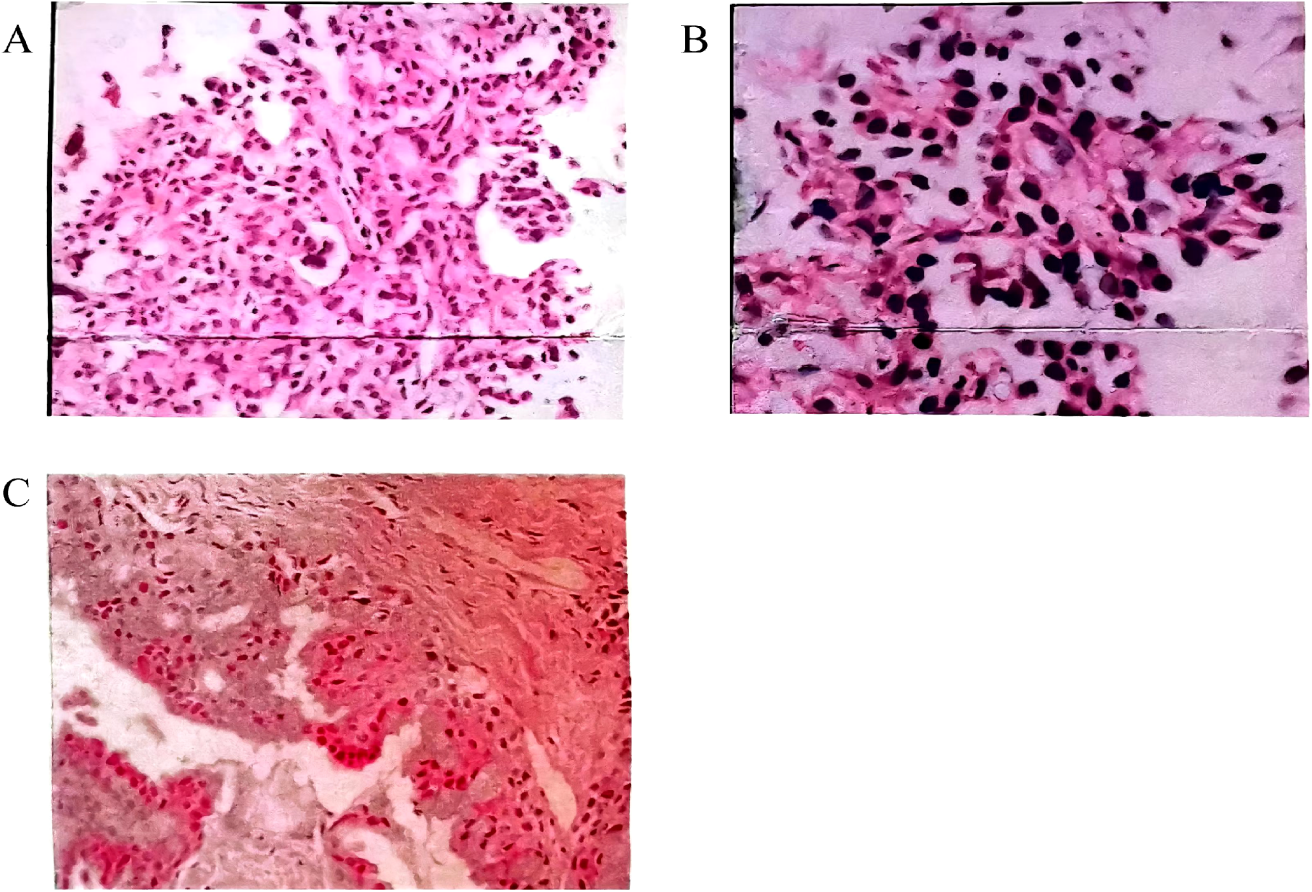
**(A, B)** Pathological examination of percutaneous lung biopsy on May 23, 2002. Microscopic analysis revealed marked alveolar epithelial hyperplasia, with bulbous cells adhering to the alveolar walls, displaying a tendency to form papillary structures. Due to the small tissue sample, a fine bronchioloalveolar carcinoma could not be excluded. **(A)** Hematoxylin and eosin (HE) staining (×200); **(B)** HE staining (×400). **(C)** HE staining from March 2016 (×200). Microscopic examination identified epithelial cell nests within fibrous tissue. Two distinct cell types were observed in the nests: mucus-secreting cells and intermediate epithelial cells. Both cell types exhibited mild morphological changes and subtle anisotropy. Nuclear pleomorphism was rare, and mucus accumulation was noted in some areas. Immunohistochemical staining: CK7 (+), TTF1 (+), p63 (+), Ki-67 (~2%).

A 44-year-old male presented with a 20-year history of intermittent cough, sputum, and hemoptysis, with a recurrence of abdominal pain over the last two days. In May 2002, the patient first experienced unexplained cough, sputum, and hemoptysis, with approximately 100 mL of blood in the sputum. He had a smoking history, which began at the age of 18 with a daily consumption of one pack. Between the ages of 24 and 44, he experienced intermittent smoking, with periods of abstinence during symptomatic flare-ups and reduced to half a pack per day during periods of symptom stability. His cumulative smoking exposure is calculated to be 15 pack-years. The patient denies any familial history of malignancies or hereditary cancer syndromes, such as Lynch syndrome or Li-Fraumeni syndrome. He is an only child, and both his parents, as well as his extended family (including uncles, aunts, and children), are currently healthy and alive. Furthermore, there is no history of toxic exposure, allergies, or tuberculosis. Chest X-ray suggested pulmonary metastasis, and a PET-CT scan from an external hospital on May 8, 2002, showed multiple nodular shadows of varying sizes in the left lung, some with calcifications and increased metabolic activity, suggestive of granulomatous lesions or tumors (no image available). Bronchoscopy revealed unobstructed airways with no new growth or stenosis, and the brush smear was negative for tumor cells or acid-fast bacteria. Percutaneous lung biopsy showed alveolar epithelial hyperplasia, with bulbous cell adherence to the alveolar walls and a tendency to form papillae (**Figures 1A, B**). Given the small tissue sample, bronchioloalveolar carcinoma could not be ruled out. Surgical biopsy was recommended but declined by the patient. After symptomatic treatment, his symptoms improved, and he was discharged, continuing with Chinese medicine.

On August 5, 2002, a chest CT scan revealed multiple dense masses of various sizes in the left lung, with a maximum diameter of approximately 2.5 cm. The lesions had clear margins and some calcifications, but the patient declined further diagnostic procedures. A follow-up CT scan on September 27, 2005, showed an increase in size of the mass in the posterior segment of the left upper lobe and patchy opacities in the right upper lobe (no image available). Despite further symptomatic treatment, the patient’s condition was not addressed with standardized treatments. From October 2015 to March 2016, the symptoms worsened, prompting visits to multiple large hospitals in China. Tumor markers were elevated, including squamous cell carcinoma antigen (13 ng/mL), carcinoembryonic antigen (17.93 ng/mL), glycoprotein antigen 125 (434.4 U/mL), and cytokeratin 19 fragment (4.42 ng/mL). Chest CT on October 13, 2015, showed the left upper lobe mass had increased in size, and new lesions appeared in the right upper lobe with enlarged mediastinal lymph nodes. A PET-CT on November 25, 2015, suggested possible malignant transformation of the left upper lung lesion, with increased metabolic activity in the left hilar lymph nodes. Bronchoscopy was unremarkable, and tuberculosis was ruled out.

In March 2016, a CT-guided percutaneous lung biopsy revealed epithelial nests containing mucous and intermediate cells, indicative of low-grade PMEC. Immunohistochemical staining was positive for CK7, TTF-1, p63, and Ki-67 (2%+) (**Figure 1C**). Given extensive involvement of both lungs, surgery was not recommended. The patient declined genetic testing and was given symptomatic treatment.

A comparison of chest CT from September 30, 2020, and October 13, 2015, showed slight progression of pulmonary lesions and the development of new cavities in the left lung. PET-CT on May 5, 2022, indicated multiple lesions in both lungs with increased metabolic activity, and mediastinal lymph nodes exhibited high SUVmax values. Further bronchoscopy in July 2022 revealed thickening of the left upper bronchial mucosa, with a biopsy confirming mucinous epithelium.

**Figure 2:**
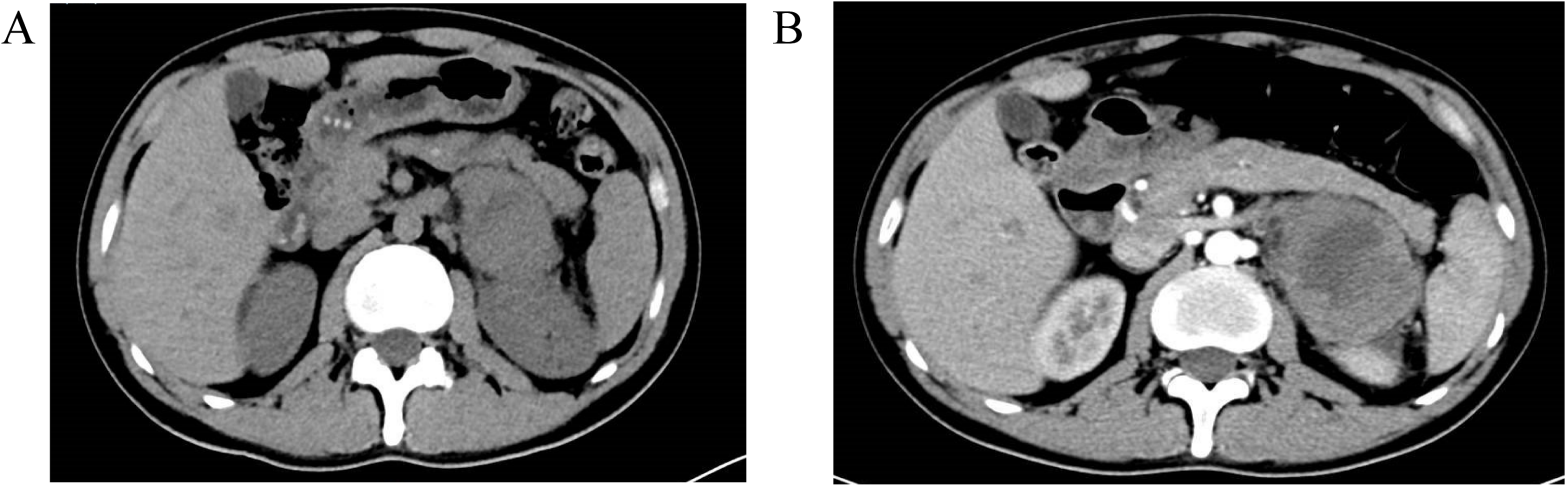
Abdominal CT scan with contrast enhancement on April 2, 2023 revealed a mass in the left adrenal region measuring 58×47×68 mm, with a small amount of surrounding exudate. Part of the mass was poorly delineated from the left kidney. Contrast enhancement showed inhomogeneous enhancement, suggesting a possible left adrenal metastasis. Multiple small low-density areas were observed in the liver, with no significant enhancement on the contrast scan, likely indicating hepatic cysts. The size and density of the gallbladder, spleen, pancreas, and bilateral kidneys were normal. No enlarged lymph nodes were identified in the abdominal cavity or retroperitoneal region, and no peritoneal effusion was present. Additionally, the lumbar vertebrae (L3) showed hyperdense, nodular lesions, and partial destruction of the lumbar vertebrae and pelvic bones, raising suspicion for metastatic tumors. **(A)** Plain scan; **(B)** Enhanced scan.

**Figure 3:**
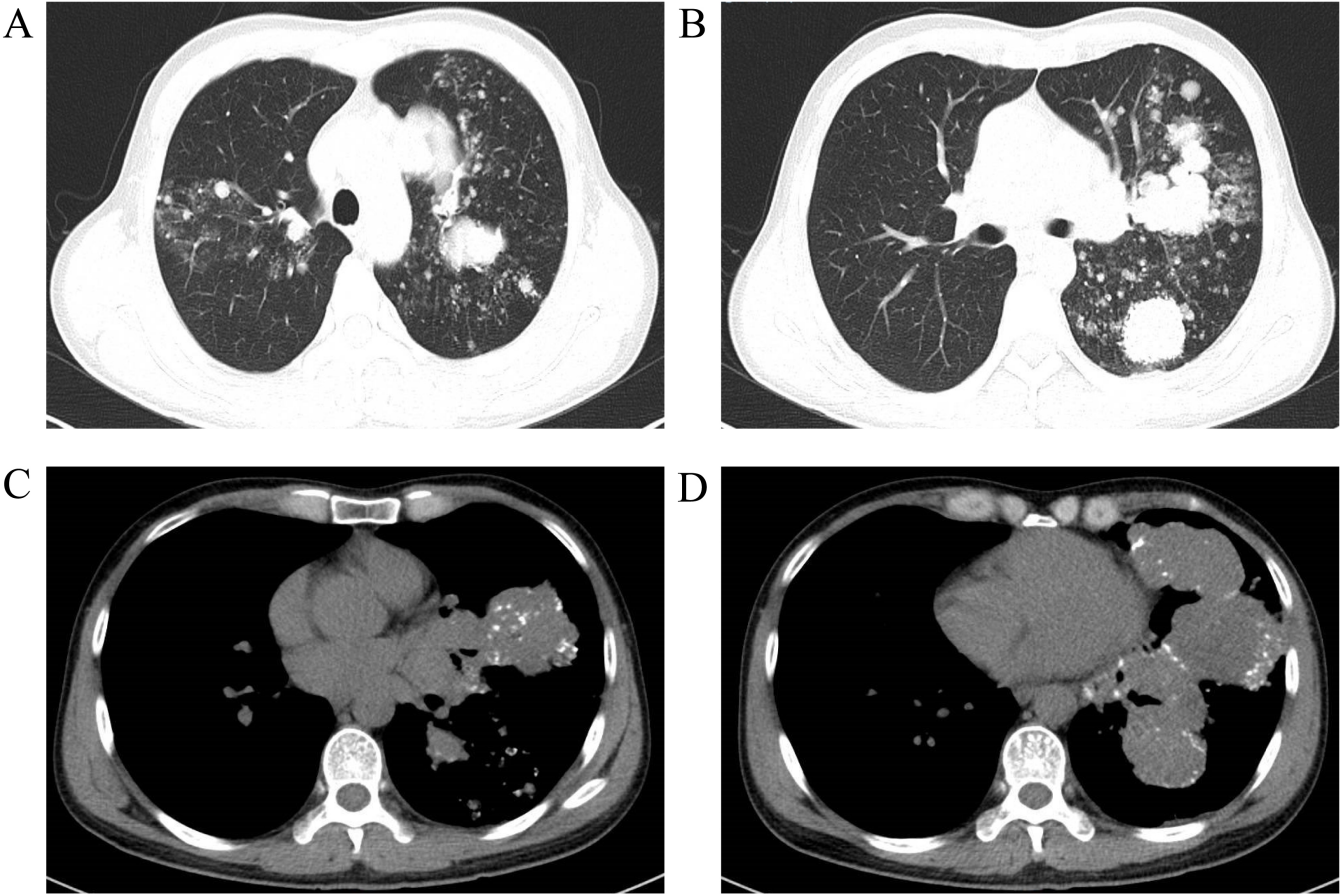
Chest CT scan with contrast enhancement on April 4, 2023 revealed multiple speckled, nodular, and clustered high-density shadows in both lungs, with pronounced uneven enhancement and well-defined margins on the contrast-enhanced images. The largest lesion, located in the upper lobe of the left lung, measured approximately 71×70×56 mm, exhibiting inhomogeneous density and multiple calcified foci within the lesion. The trachea and bronchi of each lobe were patent. Additionally, multiple enlarged lymph nodes were observed in the mediastinum and hilum. The findings suggest the presence of multiple lesions in both lungs, raising suspicion for malignancy, possibly multicentric lung cancer, or lung cancer in the upper lobe of the left lung with bilateral metastases. Other differential diagnoses include a malignant tumor of vascular origin. Compared to the CT scan from March 27, 2022, there are more lesions present in both lungs, as well as more prominent lymph node enlargement in the mediastinum and hilum. **(A, B)** Lung window; **(C, D)** Mediastinal window.

By early 2023, the patient experienced worsening symptoms including shortness of breath, fatigue, weight loss, and decreased physical activity. On April 2, 2023, he developed persistent, distending abdominal pain. A CT scan of the abdomen revealed a mass in the left adrenal gland (58 × 47 × 68 mm) with uneven enhancement, suggesting metastasis. Additional findings included multiple bone metastases, notably in the L3 vertebral body and pelvis (**Figure 2**). A chest CT on April 4, 2023, revealed multiple high-density nodules in both lungs, with an uneven enhancement pattern and foci of calcification (**Figure 3**). Pulmonary function tests indicated moderate restrictive ventilatory dysfunction.

**Figure 4:**
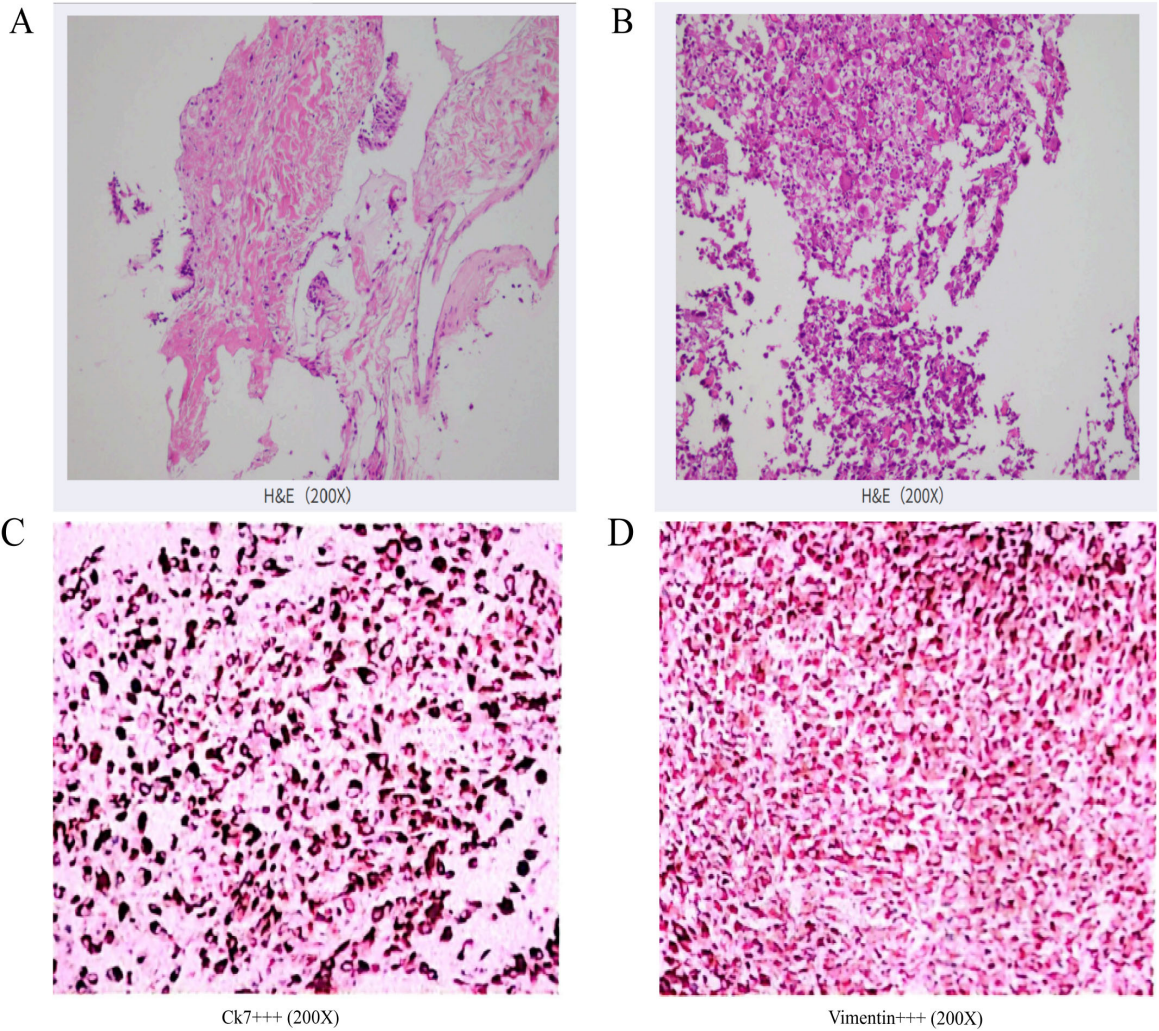
**(A)** HE staining (×200): Percutaneous lung biopsy revealed low-grade mucoepidermoid carcinoma in the left lung. Immunohistochemical staining: CK (+), CK5/6 (+), CK7 (+), Napsin-A(-), P40 (+), p63 (+), TTF-1 (-). **(B)** HE staining (×200): Pathological examination of the adrenal biopsy suggests a malignant tumor. Based on the histological pattern and immunohistochemical results, sarcomatoid carcinoma is strongly suspected. Clinical correlation is essential to determine the primary site of the tumor. Immunohistochemical results: Tumor cells show focal positivity for CK (+), strong positivity for CK7 (+++), strong positivity for Vimentin (+++), scattered positivity for CD5/6 (+), weak scattered positivity for P40 (+), occasional weak or negative CK5/6 (-/+), Ki-67 proliferation index approximately 60% (+), negative for CgA(-), inhibin (-), LCA (-), Melan-A (-), Syn (-), and TTF-1 (-). **(C)** Immunohistochemistry of adrenal puncture tumour tissue (×200): CK7 (++++); **(D)** Adrenal puncture tumour tissue immunohistochemistry (×200): Vimentin (++++).

On April 14, 2023, a CT-guided percutaneous lung biopsy confirmed a diagnosis of low-grade mucinous epithelioid carcinoma. Immunohistochemistry showed positivity for CK, CK5/6, CK7, and P40, with negativity for napsin-A, p63, and TTF-1 (**Figure 4A**). On April 19, 2023, a CT-guided biopsy of the left adrenal gland showed a sarcomatoid carcinoma (SC). Immunohistochemical staining was positive for CK7, vimentin, P40, and Ki-67 (60%), and negative for other markers, including TTF-1 (**Figures 4B–D**). Molecular testing using next-generation sequencing (NGS) revealed BRAF p.V600E mutations in lung, adrenal, and plasma samples.

Over the 20-year period from 2002 to 2022, the pulmonary lesions remained generally stable, with progression being exceptionally slow. In early 2023, the patient experienced an exacerbation of symptoms, and by April 2023, the condition began to worsen. The final diagnosis was low-grade PMEC (c-T4N2M1c, stage IVb, BRAF p.V600E) with metastatic adrenocortical SC and multiple bone metastases. The patient’s physical status score (PS) was 2. Despite treatment with dabrafenib + trametinib and radiotherapy to the left adrenal gland, symptoms did not improve, and the patient’s condition deteriorated rapidly. He passed away in early September 2023.

## Discussion

PMEC is a relatively rare malignancy, first described by Smetana et al. in 1952 (1). It is an epithelial tumor composed of mucus-producing cells, epidermoid cells, and intermediate cells, arranged in solid, glandular, or cystic patterns. PMEC accounts for approximately 0.1–0.2% of all lung cancers in the literature. SC, characterized by bidirectional differentiation into both epithelial and mesenchymal tissue, is a rare tumor often found in the respiratory tract, gastrointestinal tract, and breast (2). It is even more uncommon in the urinary tract and adrenal glands (3). ASC is a particularly rare malignancy, first described by Collina et al. in 1989 (4). To date, only a small number of cases have been reported worldwide, all as individual case reports. There are no documented cases of pulmonary mucoepidermoid carcinoma coexisting with ASC. The combination of low-grade PMEC and metastatic ASC is exceedingly rare in clinical practice, presenting challenges in diagnosis and treatment. The rapid deterioration of the patient’s condition, difficulty in treatment, and poor prognosis make this clinical scenario especially challenging.

A search of the PubMed database for literature between 1949 and March 2024 using the keywords “lung or bronchial mucoepidermoid carcinoma” and “adrenal sarcomatoid carcinoma” failed to retrieve any relevant studies. Similarly, searches of the Chinese Biomedical Literature Service System (SINOMED), China National Knowledge Infrastructure (CNKI), and Wanfang Data, using the same keywords, yielded no relevant results. Thus, to our knowledge, this is the first reported case about the combination of pulmonary mucoepidermoid carcinoma and ASC.

PMEC does not have specific clinical manifestations. The clinical symptoms primarily result from bronchial irritation and obstruction, and in some cases, there may be no symptoms at all. Common symptoms include cough and sputum production, with approximately one-third of cases associated with hemoptysis, often manifesting as blood in the sputum. Shortness of breath and chest pain can also occur. The time between symptom onset and seeking medical attention is typically 1-2 months. The patient’s symptoms often fluctuate, with intermittent bouts of cough, sputum, and blood in the sputum. These symptoms may range from mild to severe and tend to worsen with colds or physical exertion. Notably, the disease can progress over a long period—up to 20 years in this case, which is rare in clinical practice.

CT imaging plays a critical role in the diagnosis of both PMEC and adrenal tumors. PMEC most commonly occurs in the large airways, which contain abundant mucous glands, but it is rare in the trachea. It typically presents as a polypoid mass protruding into the lumen, while peripheral forms are less common. The size of the lesions varies, with shapes ranging from ovoid to lobulated. The tumor density is uneven, typically ranging from low to medium, and approximately half of the lesions exhibit speckled or granular calcifications. In this case, the PMEC was peripheral, with the main lesions located in the upper left lobe. The CT scan revealed multiple speckled, nodular, and mass-like high-density shadows with clear edges. The lesion density was uneven, with multiple calcific foci visible and uneven enhancement observed on contrast-enhanced scanning. Over the past 20 years, the lesions gradually and slowly increased in size, with very prominent calcifications. Given the patient’s long disease course, poor local blood supply, and the possibility of dystrophic calcification or incomplete absorption of mucus secreted by mucous cells, calcium salt deposition likely contributed to the observed calcifications. This is consistent with the low-grade malignant type of PMEC, which is rich in mucous cells, making calcification more prominent. The vascular distribution in low-grade versus high-grade PMEC differs significantly, and the CT enhancement patterns also vary, which can help distinguish between high- and low-grade PMEC. Enhanced chest CT can thus provide valuable information to differentiate between these two grades of PMEC. The adrenal gland is highly vascularized and is a common site for metastasis from malignant tumors. When diagnosing lung cancer, routine abdominal CT scans should be performed to assess for potential distant metastases, particularly to the adrenal glands. Metastatic adrenal tumors are more common than primary adrenal tumors. When a space-occupying lesion is identified in the adrenal gland in the context of a primary tumor, adrenal metastasis should be strongly considered, especially if the lesion is bilateral. However, in clinical practice, adrenal metastases are most often unilateral. The size of adrenal metastatic tumors can vary widely, and enhanced CT scans typically show progressive delayed enhancement, with marked enhancement during the arterial phase and persistent enhancement in the portal venous phase. Imaging of ASC is nonspecific. CT often reveals a mass with irregular borders in the adrenal region, with inhomogeneous annular enhancement in bilateral lesions. In this case, the CT scan showed a mass in the left adrenal region with unclear edges, and the enhanced scan demonstrated inhomogeneous enhancement, consistent with adrenal metastasis.

Pathological biopsy remains the gold standard for diagnosing both PMEC and ASC. PMEC is classified into low-grade and high-grade based on the degree of cell differentiation and the ratio of cell components (5). Low-grade PMEC is the most common, accounting for approximately 75-80% of cases, while high-grade PMEC is rare. High-grade PMEC is typically characterized by a predominance of intermediate and squamous cells, with a small amount of mucus. These tumors exhibit deeply stained cytoplasm, atypical nuclei, active mitosis, a high nuclear-cytoplasmic ratio, and common necrosis with evident atypia. In cases with small biopsies or morphologically atypical PMEC, immunohistochemistry is essential for diagnosis. Immunohistochemical markers are crucial for pathological typing and differential diagnosis. In this case, the immunohistochemical profile was typical for low-grade PMEC, showing negative TTF-1, positive CK7, positive CK, negative Napsin-A, and positive P40. Huo et al. also found that PMEC is consistently negative for TTF-1 (6). ASC consists of both carcinoma and sarcomatous components, with the sarcomatous component typically comprising more than 50% of the tumor (7). The sarcomatous components often include leiomyosarcoma, osteosarcoma, and chondrosarcoma. Under light microscopy, ASC is characterized by spindle cells arranged in intertwined bundles, significant cellular pleomorphism, and active mitosis. Immunohistochemistry reveals expression of mesenchymal markers such as vimentin, as well as epithelial markers like cytokeratin and EMA. In this case, the immunohistochemical analysis showed that both the cancerous and sarcomatoid components expressed CK (+) and vimentin (+). The presence of sarcomatoid components and a high Ki-67 index indicate the tumor’s aggressive nature and poor prognosis (3). In this case, the patient had a 20-year history of low-grade PMEC, with a rapid emergence of an adrenal mass. The pathology and immunohistochemistry of the percutaneous biopsy suggested ASC, which is known for its potential to metastasize to the adrenal glands. Pathological biopsy is an invasive procedure, and it is challenging to biopsy both the primary lesion and metastatic lesions in clinical practice. Few studies have explored whether the pathological type of the primary tumor is consistent with that of the metastatic lesion. Due to tumor heterogeneity, the biological behavior of the primary and metastatic lesions may differ. An 11-month-old PET-CT scan showed only lung lesions and did not detect any abdominal or adrenal involvement. In this case, the possibility of PMEC metastasizing to the adrenal glands in the form of SC is considered high. Although low-grade PMEC is typically indolent and associated with a good prognosis, this case presented a rare and unusual clinical course, with the disease progressing over 20 years. The patient’s condition worsened after a COVID-19 infection three months ago, with the development of adrenal and bone metastases. The adrenal metastases showed a change in biological behavior, becoming highly malignant and invasive, which contributed to the poor prognosis. Genetic mutations are common in PMEC, and in this case, molecular typing of the lung tumor tissue, adrenal tumor tissue, and plasma samples all revealed the presence of BRAF p.V600E, AKT1, and NFE2L2 mutations. These mutations were consistent across all tested tissues. Based on these findings, along with clinical considerations, the adrenal lesions were diagnosed as metastases from the low-grade PMEC, evolving into SC.

Histopathological examination is the gold standard for determining whether a tumor is primary or metastatic and forms the basis for clinical management. Tumor metastasis often involves epithelial-mesenchymal transition (EMT), which alters cell morphology and extracellular matrix composition, facilitating cell migration and invasion. During EMT, tumor cells transition from an epithelial-like to a mesenchymal phenotype. This transition is marked by a decrease in the epithelial marker E-cadherin and an increase in the mesenchymal markers Vimentin and N-cadherin. EMT results in the enhancement of several malignant tumor cell features, such as poor differentiation, invasiveness, resistance to apoptosis, and chemotherapy resistance, which promote the spread of the primary tumor. However, these changes can be difficult to detect through conventional pathological morphological examination and immunohistochemical marker testing. A significant proportion of tumors are poorly differentiated or undifferentiated, lacking the characteristics necessary for histological classification based on the origin of tumor cells. Immunohistochemistry can help identify the primary site of some tumors, but the detection rate remains limited. Studies have shown that the gene expression profiles of metastatic tumors differ from those of the tissue at the metastatic site, but are more similar to the profiles of the tissue at the primary site. This suggests that tumors retain the genetic expression characteristics of their tissue of origin throughout their development, growth, and metastasis. Molecular genetic testing, when compared to imaging and histopathological methods, offers significant advantages in terms of sensitivity, specificity, and objective result interpretation. It has increasingly been used as an adjunct to diagnose the primary site of tumors in several developed countries, particularly in Europe and the United States (8–12). For instance, the Cancer Origin biochip method has an accuracy rate of up to 85%, while CancerTYPE ID, using RT-PCR, achieves an accuracy rate of up to 87%. The Tissue of Origin test, using a biochip method, boasts an accuracy rate of up to 89%. This test, which is US FDA-approved, can identify 15 cancer types, including colorectal cancer, pancreatic cancer, non-small cell lung cancer, breast cancer, gastric cancer, kidney cancer, liver cancer, ovarian cancer, soft tissue sarcoma, non-Hodgkin’s lymphoma, thyroid cancer, prostate cancer, melanoma, bladder cancer, and testicular germ cell cancer. The test covers more than 95% of solid tumors, whether primary or metastatic. However, in this case, because the patient had SC of the adrenal gland, it was uncertain whether this method could conclusively identify the origin of the tumor. As a result, the test was not performed.

In this case, NGS of the lung tumor tissue, adrenal tumor tissue, and plasma revealed positive BRAF p.V600E, AKT1, and NFE2L2 mutations, which are common genetic mutations. BRAF mutations are known oncogenes, encoding a serine/threonine kinase that plays a critical role in regulating the mitogen-activated protein kinase (MAPK) cascade, which in turn modulates the expression of genes involved in various cellular functions. Germline BRAF mutations are found in the majority of melanomas, thyroid cancers, and histiocytic tumors, as well as in a small proportion of lung and colorectal cancers. Somatic BRAF mutations are commonly observed in melanoma, thyroid cancer, and lung cancer (13). The most common somatic mutation, p.V600E, leads to constitutive activation of BRAF kinase activity, allowing it to signal independently of upstream activation by RAS. As a key component of the MAPK/ERK signaling pathway, BRAF mutations drive sustained pathway activation, promoting cell proliferation, invasion, and resistance to apoptosis, which may accelerate the malignant transformation of tumors. Furthermore, BRAF mutations can induce genomic instability, increasing tumor heterogeneity. For instance, in gliomas, BRAF fusion variants have been found to cause complex structural rearrangements (e.g., selective splicing, frameshift rearrangements), potentially promoting phenotypic transformation of tumor cells. Sarcomatoid cancers, known for their high degree of heterogeneity, may undergo EMT driven by BRAF mutations, leading to the transformation of MEC into SC. BRAF mutations may also cooperate with other driver mutations (e.g., TP53, KRAS) or epigenetic alterations, suggesting a potential synergistic effect (14). However, the exact mechanisms and their interactions with other genetic changes require further exploration through molecular network studies and more comprehensive basic and clinical research. BRAF mutations are associated with poor prognosis in cancer patients. For malignancies with BRAF mutations, treatment typically involves BRAF inhibitors such as dabrafenib, alone or in combination with MEK inhibitors like trametinib. In BRAF-mutant melanoma, monotherapy with BRAF inhibitors has shown an overall survival (OS) of 13.6 months, progression-free survival (PFS) of 6.9 months, and an objective response rate (ORR) of 48%. When combined with MEK inhibitors, the OS extends to 22.3–33.6 months, PFS to 11.1–14.9 months, and ORR increases to 63–70%. In papillary thyroid cancer, the incidence of BRAF mutations ranges from 45% to 50.9%, with dabrafenib and trametinib combination therapy yielding an OS of 14.5 months, PFS of 6.7 months, and ORR of 56%. In non-small cell lung cancer, where BRAF mutations are found in 3% of cases, the combination therapy results in PFS of 9.0–14.6 months and an ORR of 63.2%–64% (15). In the present case, the limited efficacy of dabrafenib combined with trametinib could be attributed to the patient’s prolonged pulmonary disease history, which lasted 20 years, leading to poor treatment response. Moreover, the tumor type underwent transformation following metastasis, with increased tumor heterogeneity, which may be linked to the more aggressive nature of the SC after transformation. Although the patient’s BRAF mutation was detected through NGS, the mutation burden was relatively low, both in the primary lung tumor and the adrenal metastatic tissue (~10%), and even lower in plasma (1.58%), which may contribute to the suboptimal treatment outcome. The AKT signaling pathway plays a pivotal role in tumorigenesis and progression in various cancers. AKT1 is an oncogene that encodes a serine/threonine protein kinase, serving as a key downstream regulator in the PI3K signaling pathway (16). AKT1 activates several downstream substrates, including GSK3, FOXO, and mTORC1, which are crucial for cell survival, proliferation, and metabolism. The activity of AKT1 is negatively regulated when the PI3K signaling is terminated by the phosphatase activity of PTEN. AKT1 is typically activated in cancer through the activation of the PI3K pathway or through the inactivation of PTEN. Somatic mutations in AKT1 have been identified in various human cancers, including breast cancer, colorectal cancer, and ovarian cancer (17). In this case, AKT1 was detected, suggesting its potential involvement in the pathogenesis and progression of PMEC. NFE2L2, also known as NRF2, is a transcription factor critical for the oxidative stress response. It belongs to the Cap’n’Collar (CNC) subfamily of bZIP transcription factors and regulates genes containing antioxidant response elements (ARE) in their promoters. The proteins encoded by these genes are largely involved in damage and inflammation responses. NRF2 plays a dual role in cancer progression. It is widely expressed in various tissues, with the highest expression levels in adult muscle, kidney, lung, liver, and fetal muscle. A 2006 study identified mutations in KEAP1 in non-small cell lung cancer (NSCLC), leading to sustained elevated levels of NRF2, and first proposed that NRF2 might contribute to cancer progression and chemotherapy resistance. Subsequent studies have shown that excessive activation of the NRF2 pathway creates an environment that not only supports normal cell survival but also promotes the survival of cancer cells, protecting them from oxidative stress, chemotherapy drugs, and radiotherapy. Experiments have demonstrated that Nrf2 activation of antioxidant enzymes (e.g., Trx, Prx, and GCL) drives MCF-7 cells to develop resistance to tamoxifen. Additionally, Nrf2 can activate the expression of multidrug resistance proteins (MRPs), thereby reducing the accumulation of anticancer drugs in cancer cells. Several phase II enzymes regulated by Nrf2 have also been shown to confer chemotherapy resistance.

Considering that the lung lesions appeared first, followed by the appearance of adrenal lesions after progression, a comprehensive analysis suggested that the adrenal lesions were metastatic. Clinically, it is rare for the type and biological behavior of metastatic lesions to change, which makes the identification of such lesions challenging. Zhao et al. (18)reported a rare case of metastatic adenocarcinoma of the lung originating from a low-grade appendiceal mucinous tumor (LAMN), where NGS was also used to elucidate the relationship between the two tumors. Sequencing revealed common mutations, including KRAS (p.G12D), GNAS (p.R201H), and BRAF (p.R735Q), suggesting that the lung tumor was a metastasis of the LAMN. This case shares similarities with the current one, highlighting the potential of NGS to clarify the relationship between primary and metastatic lesions despite differing tumor characteristics (18).

There is no standard treatment for this condition due to the lack of relevant reports. However, treatment strategies for advanced lung cancer with adrenal metastases may serve as a reference. This case involves advanced low-grade PMEC with adrenal and bone metastases, and molecular typing of lung tissue, adrenal tissue, and plasma revealed positive BRAF p.V600E mutations, with abundances of 10.74%, 13.03%, and 1.58%, respectively. The final diagnosis was low-grade PMEC (c-T4N2M1c, stage IVb) with BRAF p.V600E mutation, ASC metastasis, and multiple bone metastases, with a physical status score (PS) of 2.The patient received molecular targeted therapy with dabrafenib and trametinib, combined with radiotherapy to the left adrenal area. Unfortunately, the treatment response was poor. This is likely due to several factors, including the presence of sarcomatoid components in the adrenal metastasis, the high Ki-67 marker index indicating high tumor proliferation, the long disease course, the high tumor burden, and the patient’s low systemic immunity. These factors may have contributed to the limited effectiveness of the targeted therapy. The findings suggest that PMEC carries driver gene mutations, but the response to molecular targeted therapy can vary between individuals, depending on the tumor characteristics and genetic mutations. Future research should focus on identifying patient populations that are more likely to benefit from molecular targeted therapies for advanced PMEC. Further studies are needed to understand the heterogeneity of treatment responses and to refine therapeutic strategies for this rare and complex disease.

Currently, there is a lack of large-scale, prospective clinical trial data on immunotherapy for BRAF V600E-mutant NSCLC. A retrospective analysis revealed that NSCLC patients with BRAF V600E mutations (10 cases) had a lower tumor mutational burden (TMB) compared to non-V600E-mutant NSCLC patients (42 cases), and showed a lower ORR to immunotherapy (11% versus 23%, respectively). Additionally, the median duration of response to immunotherapy was shorter in both BRAF V600E-mutant and non-V600E-mutant NSCLC patients (1.3 months vs. 2.2 months, respectively). A multicenter retrospective study in China analyzed data from 4,178 NSCLC patients treated with immune checkpoint inhibitors (ICIs) alone, of whom 6.1% had BRAF mutations. The study explored the efficacy of immunotherapy in BRAF-mutant NSCLC patients and found that the median OS of the BRAF V600E group (5 cases) was significantly lower than that of the non-V600E BRAF group (21 cases) (5 months vs. 14 months, P=0.017). Another retrospective analysis investigating the efficacy of immunotherapy in advanced NSCLC with driver gene mutations included 551 patients, of which 43 had BRAF mutations. The ORR for BRAF-mutant patients was 24%, with a median PFS of 3.1 months and a median OS of 13.6 months. However, the survival benefit differences between BRAF V600E-mutant (17 cases) and other BRAF mutation types (18 cases) were not statistically significant (19, 20).

## Conclusion

PMEC combined with ASC metastasis is a rare clinical occurrence. The clinical features and CT manifestations are nonspecific, making diagnosis challenging. Pathological diagnosis remains the gold standard for identifying this disease. The pathological type, biological behavior, and invasiveness can differ between primary and metastatic lesions. In cases where identification is difficult, NGS can be used to clarify the relationship between the primary and metastatic lesions. Although the pathological type, biological behavior, and invasiveness may vary, the genetic mutation status can remain consistent. It is important to note that the inability to conduct immunohistochemical testing for adrenal-specific markers to confirm the adrenocortical origin of ASC represents a limitation of this study. However, it is difficult to biopsy both the primary and metastatic lesions separately in clinical practice.

Whether this case represents an individual occurrence or a more common phenomenon requires further clinical data. In future clinical practice, if the patient’s condition permits, biopsies of both the primary and metastatic lesions should be considered, especially when there is clinical suspicion of differing biological behavior, distant metastasis from a small primary lesion, poor treatment response, or discrepancies in treatment efficacy. Our study provides valuable insights for diagnosing rare cases of coexisting tumors, such as the need for multi-lesion biopsies, and for selecting appropriate targeted therapies. Additionally, the study highlights the heterogeneity of BRAF-mutated tumors and the role of epithelial-to-mesenchymal transition (EMT) in the phenotypic transformation of metastases, warranting further investigation through basic research. Accumulating more clinical experience and data will enable more precise and personalized treatment strategies for such complex cases.

## Data Availability

The original contributions presented in the study are included in the article/supplementary material. Further inquiries can be directed to the corresponding author.

## References

[1] Rauniyar R Sharma A Thapaliya P Sapkota R . Primary pulmonary endobronchial mucoepidermoid carcinoma treated with sleeve resection in a 23-year-old woman: case report. Respirol Case Rep. (2025) 13 4:e70173. doi: 10.1002/rcr2.70173, PMID: 40231311 PMC11994294

[2] Zhu C Zheng A Mao X Shi B Li X . Primary adrenal sarcomatoid carcinoma metastatic to the lung: Case report and review of the literature. Oncol Lett. (2016) 11:3117–22. doi: 10.3892/ol.2016.4342, PMID: 27123074 PMC4841111

[3] Ahmed AA Thomas AJ Ganeshan DM Blair KJ Lall C Lee JT . Adrenal cortical carcinoma: pathology, genomics, prognosis, imaging features, and mimics with impact on management. Abdom Radiol (NY). (2020) 45:945–63. doi: 10.1007/s00261-019-02371-y, PMID: 31894378

[4] Collina G Maldarizzi F Betts CM Eusebi V . Primary sarcomatoid carcinoma of the adrenal gland. First case report. Virchows Arch A Pathol Anat Histopathol. (1989) 415:161–7. doi: 10.1007/BF00784354, PMID: 2472700

[5] Hanif Khan A Faisal M Mohd Ali R Abdul Rahaman JA . Resolution of asthmatic symptoms following successful endoscopic resection of tracheal mucoepidermoid carcinoma. BMJ Case Rep. (2019) 12(1):e226202. doi: 10.1136/bcr-2018-226202, PMID: 30659001 PMC6340577

[6] Huo Z Wu H Li J Li S Wu S Liu Y . Primary pulmonary mucoepidermoid carcinoma: histopathological and moleculargenetic studies of 26 cases. PloS One. (2015) 10:e0143169. doi: 10.1371/journal.pone.0143169, PMID: 26575266 PMC4648574

[7] Papathomas TG Duregon E Korpershoek E Restuccia DF Van Marion R Cappellesso R . Sarcomatoid adrenocortical carcinoma: a comprehensive pathological, immunohistochemical, and targeted next-generation sequencing analysis. Hum Pathol. (2016) 58:113–22. doi: 10.1016/j.humpath.2016.08.006, PMID: 27589897

[8] Eguren-Santamaria I Sanchez-Bayona R Patiño-Garcia A Gil-Bazo I Lopez-Picazo JM . Targeted DNA sequencing for assessing clonality in multiple lung tumors: A new approach to an old dilemma. Lung Cancer. (2018) 122:120–3. doi: 10.1016/j.lungcan.2018.05.029, PMID: 30032819

[9] Mansuet-Lupo A Barritault M Alifano M Janet-Vendroux A Zarmaev M Biton J . Proposal for a combined histomolecular algorithm to distinguish multiple primary adenocarcinomas from intrapulmonary metastasis in patients with multiple lung tumors. J Thorac Oncol. (2019) 14:844–56. doi: 10.1016/j.jtho.2019.01.017, PMID: 30721797

[10] Zhao Y Wu J Pei F Zhang Y Bai S Shi L . Molecular typing and clinical characteristics of synchronous multiple primary colorectal cancer. JAMA Netw Open. (2022) 5:e2243457. doi: 10.1001/jamanetworkopen.2022.43457, PMID: 36416825 PMC9685491

[11] Liang Z Zeng G Wan W Deng B Chen C Li F . The unique genetic mutation characteristics based on large panel next-generation sequencing (NGS) detection in multiple primary lung cancers (MPLC) patients. Discov Med. (2023) 35:131–43. doi: 10.24976/Discov.Med.202335175.14, PMID: 37188510

[12] Lu M Zhang X Chu Q Chen Y Zhang P . Susceptibility genes associated with multiple primary cancers. Cancers (Basel). (2023) 15(24):5788. doi: 10.3390/cancers15245788, PMID: 38136334 PMC10741435

[13] Dankner M Rose AAN Rajkumar S Siegel PM Watson IR . Classifying BRAF alterations in cancer: new rational therapeutic strategies for actionable mutations. Oncogene. (2018) 37:3183–99. doi: 10.1038/s41388-018-0171-x, PMID: 29540830

[14] Mechahougui H Gutmans J Gouasmi R Smekens L Friedlaender A . BRAF targeting across solid tumors: molecular aspects and clinical applications. Int J Mol Sci. (2025) 26(8):3757. doi: 10.3390/ijms26083757, PMID: 40332392 PMC12027668

[15] Wang W Lian B Xu C Wang Q Li Z Zheng N . Expert consensus on the diagnosis and treatment of solid tumors with BRAF mutations. Innovation (Cambridge (Mass)). (2024) 5:100661. doi: 10.1016/j.xinn.2024.100661, PMID: 39529955 PMC11551471

[16] De SK . Capivasertib: first approved AKT inhibitor for the treatment of patients with breast cancer. Anti Cancer Agents Med Chem. (2025) 25:371–7. doi: 10.2174/0118715206360571241126080725, PMID: 39633517

[17] Kumar BH Kabekkodu SP Pai KSR . Structural insights of AKT and its activation mechanism for drug development. Mol Divers. (2025). doi: 10.1007/s11030-025-11132-7, PMID: 40009150 PMC12638386

[18] Zhao XY Li CQ Zhang SY Liu G . Case report: an unusual case of pulmonary metastatic adenocarcinoma from low-grade appendiceal mucinous neoplasms. Front Oncol. (2022) 12:906344. doi: 10.3389/fonc.2022.906344, PMID: 35912193 PMC9327614

[19] Mazieres J Drilon A Lusque A Mhanna L Cortot AB Mezquita L . Immune checkpoint inhibitors for patients with advanced lung cancer and oncogenic driver alterations: results from the IMMUNOTARGET registry. Ann Oncol: Off J Eur Soc Med Oncol. (2019) 30:1321–8. doi: 10.1093/annonc/mdz167, PMID: 31125062 PMC7389252

[20] Sullivan RJ Infante JR Janku F Wong DJL Sosman JA Keedy V . First-in-class ERK1/2 inhibitor ulixertinib (BVD-523) in patients with MAPK mutant advanced solid tumors: results of a phase I dose-escalation and expansion study. Cancer Discov. (2018) 8:184–95. doi: 10.1158/2159-8290.CD-17-1119, PMID: 29247021

